# Investigation of the Relationship Between Heavy Metals (Cadmium, Arsenic, and Lead) and Metallothionein in Multiple Sclerosis

**DOI:** 10.7759/cureus.66754

**Published:** 2024-08-13

**Authors:** Can Keçecioğlu, Cansu Sarıkaya, Ahmet Aydın, Mohammad Charehsaz, Hüsnü Efendi

**Affiliations:** 1 Pharmacy Services Program, Istanbul Galata University, Istanbul, TUR; 2 Neurology, Maltepe University Faculty of Medicine, Istanbul, TUR; 3 Pharmaceutical Toxicology, Yeditepe University, Istanbul, TUR; 4 Neurology, Kocaeli University, Kocaeli, TUR

**Keywords:** multiple sclerosis, metallothionein, lead, heavy metals, environmental factors, cadmium, arsenic

## Abstract

Background and aim: Multiple sclerosis (MS) is one of the most common neurological disorders. Metals are important for the maintenance and preservation of homeostasis and dysregulated metal homeostasis has an impact on neurodegeneration. Environmental factors are considered to contribute to MS risk and progression. Heavy metals such as arsenic (As), cadmium (Cd), and lead (Pb) are widely found in the environment and because of their toxic nature, they pose a great danger to human health. Metallothioneins (MTs) play important roles in metal homeostasis and detoxification of heavy metals.

Objective: The aim of this study was to investigate the relationship between levels of heavy metals (As, Cd, and Pb) and MT levels in MS patients and also to assess the oxidative stress status of patients.

Method: Fifty subjects (20 healthy subjects and 30 MS patients) were included. Demographic characteristics of the patients, plasma MT levels, blood Cd, As, and Pb levels, as well as iron (Fe), copper (Cu), and zinc (Zn) levels, were determined. Malondialdehyde (MDA) levels were investigated as a marker of oxidative stress.

Results: MT levels were slightly higher in the MS group (p > 0.05). As Cd and Pb levels were significantly higher in the control subjects. MDA levels were significantly higher in MS patients.

Conclusion: Our results support the relevance of MT and MDA levels in MS. Further clinical studies with larger cohorts will provide more insights into these factors.

## Introduction

Multiple sclerosis (MS) is one of the most common neurodegenerative conditions causing neurological disturbances in young adults [1]. The age of MS onset is between 20 and 40 years with three times higher incidence in women than men [2]. MS etiology is still unknown; however, interactions between genetic and environmental factors are considered to contribute to MS risk and progression [3,4]. Although various treatment strategies have been developed for MS, the disease can be progressive [5].

Essential elements are important factors for the maintenance and preservation of cellular homeostasis and play important roles in the regulation of intracellular processes [6]. Dysregulated essential element homeostasis has been implicated in various diseases including neurodegeneration [7]. Toxic elements, on the other hand, are ubiquitously found in the environment and due to their toxic nature [8,9] and extended biological half-life in the body [10-12], as well as the tendency to remain in the ecosystem and their movement throughout the food chain [13], they possess great danger for the human health. Exposure to toxic elements such as cadmium (Cd), lead (Pb), and arsenic (As) has been indicated to disrupt neurological and cognitive functions [14,15].

Metallothioneins (MTs) are proteins with small molecular weight and they belong to the cysteine-rich, metal-binding group of proteins. They bind to divalent metal ions with high affinity [16]. MTs play important roles in the regulation of metal homeostasis, detoxification of heavy metals, protection against oxidative stress, regulation of cell proliferation and death, and regulation of neuronal growth [17,18]. It was reported that MT-1 and MT-2 interact with osteopontin, an extracellular matrix protein with critical roles in autoimmune demyelinating diseases such as MS [19], and the interactions between two MTs and osteopontin improve tissue remodeling in encephalomyelitis [20]. MTs also play protective roles in heavy metal-induced toxicity [21-23]. Slightly elevated MT expression in inactive lesions in MS patients has been suggested to be crucial for disease remission [24]. In addition, in experimental autoimmune encephalomyelitis (EAE) in mice, MT levels were shown to be increased [24-26].

Literature on the role of heavy metals in MS is limited. A study conducted in Iran revealed that MS patients had higher blood levels of Cd, but not Pb, compared with healthy individuals [27]. In another Iranian study, blood As and Cd levels were significantly higher in MS patients than in control subjects [28]. A study conducted in Taiwan reported that soil Pb and As concentrations were positively and negatively correlated with MS incidence, respectively [29]. On the other hand, malondialdehyde (MDA), a lipid peroxidation marker [30], has been found to be significantly increased in MS patients [31,32]. Yousefi et al. reported high serum As levels, as well as MDA levels, suggesting a relationship between oxidative damage, demyelination, and neurodegeneration [33]. Against this background, the aim of the present study, conducted on MS patients in Turkey, was to investigate the possible relationship between heavy metals (As, Cd, and Pb), MTs, MDA, and MS.

## Materials and methods

Patient group and study design

The study was approved by the local ethics committee for non-invasive clinical research (approval no: KÜ GOKAEK 2017/5.22). This study was conducted at Kocaeli University, Kocaeli, Turkey, and Yeditepe University, Istanbul, Turkey. Patients were randomly selected among those who came to the neurology outpatient clinic of Kocaeli University. Blood samples taken from patients were studied at Yeditepe University. Blood samples taken from patients were studied at Yeditepe University. Fifty subjects aged between 18 and 55 (20 healthy controls and 30 MS subjects) were recruited. The MS patient group comprised patients attending to the MS outpatient clinic for routine control between July 2018 and November 2018. The members of the control group, with similar age and gender characteristics as the patient group, were selected among hospital staff and patient’s relatives willing to participate in the study. Written informed consent was obtained from all participants prior to the study.

Patients with relapsing-remitting MS (RRMS) whose last attack had occurred at least 30 days before study admission were included. Instead, patients who were diagnosed with progressive MS, experienced acute attacks, could potentially be exposed to As, Cd, or Pb through their work, or had a history of systemic or metabolic disease, or a known history of known malignancy, were excluded from the study.

Biological sample collection

Five mL blood samples were collected from the patient and control groups into the ethylene diamine tetraacetic acid (EDTA)-treated tubes (BD Vacutainer® Plus Plastic K2EDTA Tubes; 367899, BD Diagnostics, Franklin Lakes, United States). The samples were centrifuged at 4,400 rpm for 7-10 min and supernatant plasma was stored at -80°C for MT-1A and MDA analysis. For the preparation of erythrocytes, pellets were washed with 0.9% NaCl and centrifuged at 4,400 rpm for 10 min. This step was repeated two more times. After the last centrifugation, the cells were resuspended with 1 mL 0.9% NaCl, transferred to a clean tube, and mixed with 4 mL cold distilled water. The erythrocytes were then lysed by vortexing and the lysates were stored at -80°C for heavy metal analysis.

Determination of toxic and essential element levels

Toxic and essential metal levels were determined by using an inductively coupled plasma-mass spectrometer (ICP-MS; XSERIES 2, Thermo Scientific™, Waltham, United States). The parameters for running the samples were set on the device as follows: forward power = 1450 watts, nebulizer flow = 0.9 mL/min, auxiliary gas flow = 0.8 mL/min, cool gas flow = 13 mL/min, collision cell technology gas flow = 0.6 mL/min. The intensity counts per second (ICPS) was the measurement unit used for each standard and analyte.

Determination of MT levels

Plasma MT-1A and its analogs were determined by using a human MT-1A enzyme-linked immunosorbent assay (ELISA) kit (NBP2-60092, Novus Biologicals, Centennial, United States) according to the manufacturer’s instructions. The optical density (OD) of each well was determined at a wavelength of 450 nm on a multiplate reader (Multiskan Ascent, Thermo Labsystems, Vantaa, Finland) and the concentrations of the MT-1A and its analogs present in each sample were determined.

MDA determination

Lipid peroxidation was investigated by determining plasma MDA levels as described [34].

Statistical analysis

GraphPad Prism® 7.0 (GraphPad Software Inc., United States) was used for the statistical analyses The data were expressed as mean ± standard deviation (SD). Blood heavy metal level values found to be negative were excluded from the analysis. The Shapiro-Wilk normality test was used to analyze the distribution of the data. Normally distributed data were analyzed using an unpaired two-tailed Student’s t-test, and the Mann-Whitney U test was employed to analyze non-normally distributed data. Correlations between parameters were investigated by using Pearson’s correlation coefficient test. The chi-square test was used to investigate the relationship between MS risk and smoking status. Relative risk and 95% confidence interval were investigated using the Koopman asymptotic score. P < 0.05 was considered as statistically significant.

## Results

Demographic characteristics of the study population

Seventeen participants were male (healthy = 7, MS = 10), and 33 subjects were female (healthy = 13, MS = 20). Their mean age was 33.08 ± 8.3 years (healthy = 29.40 ± 3.91 years, MS group = 35.53 ± 10.30 years). The mean age of the male and female members of the control group was 28.71 ± 3.77 and 29.77 ± 4.09 years, respectively, while in the MS group, the mean age of the male and female patients was 33.80 ± 9.64 and 36.4 ± 10.75 years, respectively.

Blood As, Cd, and Pb levels

**Figure 1:**
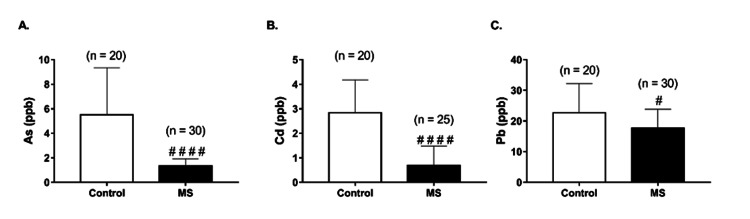
Blood arsenic (As) (A), cadmium (Cd) (B), and lead (Pb) (C) levels of control subjects and multiple sclerosis (MS) patients Data is presented as mean ± SD. Statistical analysis: Mann-Whitney U test (^#^p < 0.005 and ^####^p < 0.0001)

Mean blood As, Cd, and Pb levels were significantly lower in the MS patients compared with the control group (p < 0.0001, p < 0.0001, and p < 0.05, respectively, Figures 1A-1C). In five MS patients, blood Cd level values were found to be negative and therefore excluded. Mean blood Pb levels were significantly higher in the control subjects than in the MS patients (22.73 ± 9.444 ppb vs. 17.73 ± 6.16 ppb; p < 0.05; Figure 1C).

**Figure 2:**
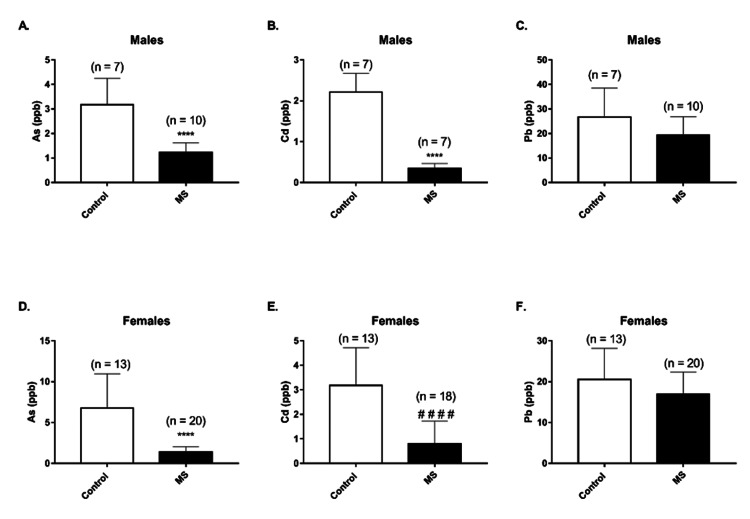
Blood arsenic (As), cadmium (Cd), and lead (Pb) levels in control subjects and multiple sclerosis (MS) patients. Data include male (A-C) and female (D-F) subjects Data is presented as mean ± SD. Statistical analysis: unpaired Student’s t-test (^****^p < 0.0001) and Mann-Whitney U test (^####^p < 0.0001)

Blood As and Cd levels were significantly higher in both male and female control subjects than in male and female MS patients (p < 0.0001; Figures 2A, 2B, 2D, 2E); however, Pb levels were similar in the control and MS groups irrespective of gender (Figures 2C, 2F).

**Figure 3:**
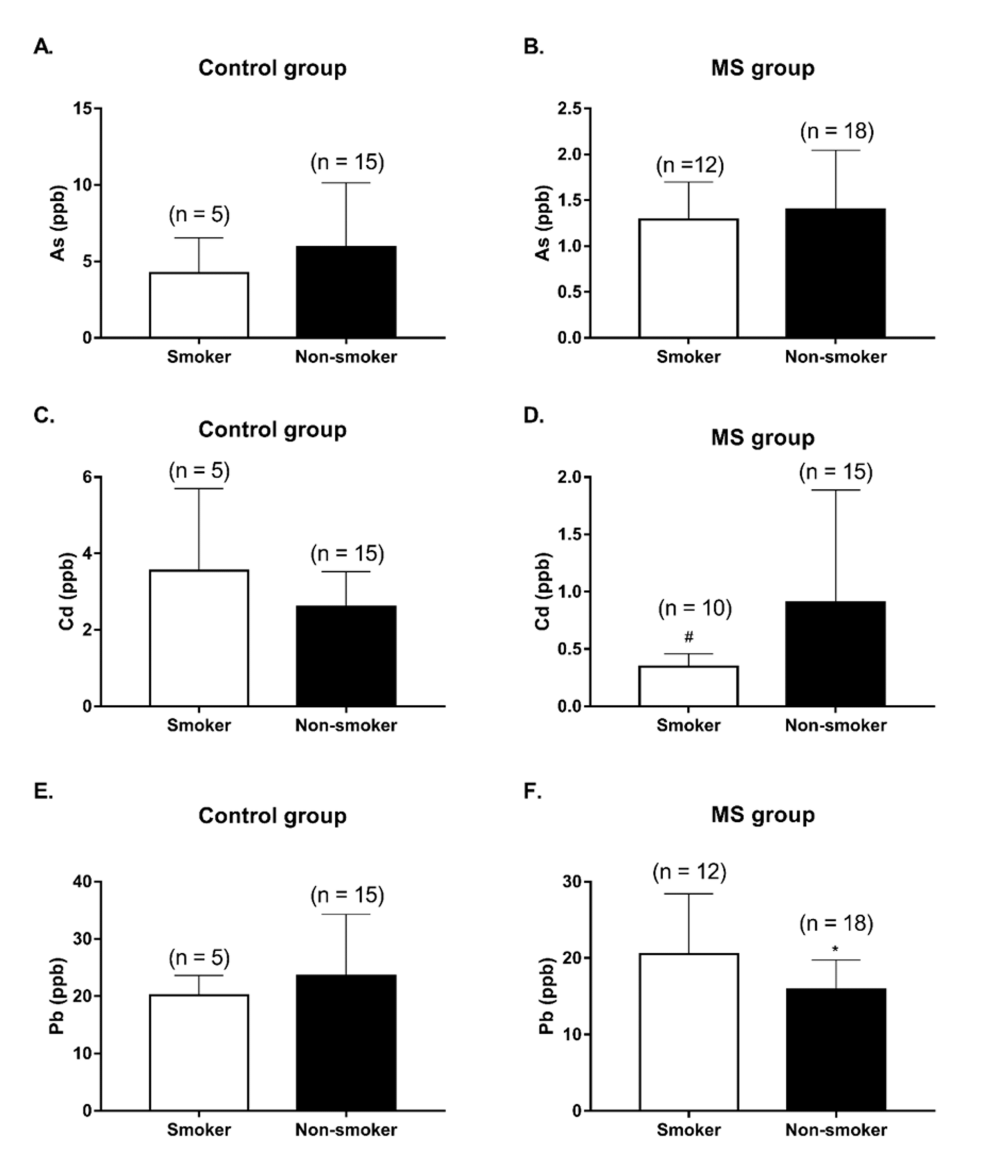
Blood arsenic (As) (A-B), cadmium (Cd) (C-D), and lead (Pb) (E-F) levels in smoker and non-smoker subjects in control and multiple sclerosis (MS) groups Data is presented as mean ± SD. Statistical analysis: unpaired Student’s t-test (^*^p < 0.05) and Mann-Whitney U test (^#^p < 0.05)

In the overall population blood As, Cd, and Pb levels between smokers and non-smokers were similar (2.157 ± 1.833 ppb vs. 3.468 ± 3.632 ppb, 1.413 ± 1.943 ppb vs. 1.754 ± 1.274 ppb and 20.43 ± 6.759 ppb vs. 19.37 ± 8.572 ppb, respectively). Moreover, both in the controls and in the MS group, blood As levels in smokers were similar to those recorded in non-smokers (Figures 3A, 3B). Instead, in the MS group, blood Cd levels were significantly lower in smokers, while no difference was found in the control group (Figures 3C, 3D); finally, blood Pb levels were significantly lower in the non-smokers in the MS group, whereas in the control group, they were similar between smokers and non-smokers (Figures 3E, 3F).

Determination of iron (Fe), copper (Cu), and zinc (Zn) levels

Fe and Zn levels were significantly higher in the control group than in the MS group (p = 0.014 and p = 0.039, respectively; Table 1), while Cu was significantly lower in the MS group compared with the controls (p = 0.036; Table 1). On analysis of the MS and control subjects divided by gender, no differences in Fe, Cu, and Zn were found between female MS and control subjects, while in male MS and control subjects, Fe, Cu, and Zn levels were significantly higher control group compared to the MS group (p = 0.008, p = 0.003, and p = 0.004, respectively; Table 1). Conversely, blood Fe, Cu, and Zn levels did not differ between the control and MS group of subjects divided by smoking status (Table 2).

**Table 1:** Blood iron (Fe), copper (Cu), and zinc (Zn) levels in the control and multiple sclerosis (MS) group of subjects ^t^: Student’s t-test; ^M^: Mann-Whitney U test

	Control (mean ± SD)	MS (mean ± SD)	p-value	Male		Female		p-value	
				Control (mean ± SD)	MS (mean ± SD)	Control (mean ± SD)	MS (mean ± SD)	Male	Female
Fe (ppm)	144.60 ± 32.34	127.90 ± 40.01	0.014^M^	153.20 ± 21.57	120.00 ± 22.19	139.90 ± 36.82	131.40 ± 46.58	0.008^t^	0.298^M^
Cu (ppm)	0.13 ± 0.03	0.125 ± 0.061	0.036^M^	0.13 ± 0.02	0.11 ± 0.02	0.13 ± 0.03	0.14 ± 0.07	0.003^t^	0.477^M^
Zn (ppm)	1.96 ± 0.52	1.70 ± 0.54	0.039^M^	2.05 ± 0.51	1.45 ± 0.37	1.92 ± 0.53	1.83 ± 0.57	0.004^M^	0.412^t^

**Table 2:** Blood iron (Fe), copper (Cu), and zinc (Zn) levels in control subjects and multiple sclerosis (MS) patients divided by smoking status ^t^: Student’s t-test; ^M^: Mann-Whitney U test

	Smoker (mean ± SD)	Non-smoker (mean ± SD)	p-value	Control		MS		p-value	
				Smoker (mean ± SD)	Non-smoker (mean ± SD)	Smoker (mean ± SD)	Non-smoker (mean ± SD)	Control	MS
Fe (ppm)	138.50 ± 29.98	132.50 ± 41.40	0.416^M^	154.60 ± 18.14	141.20 ± 35.74	131.70 ± 31.95	125.30 ± 45.30	0.438^t^	0.266^M^
Cu (ppm)	0.12 ± 0.02	0.13 ± 0.06	0.651^M^	0.13 ± 0.02	0.13 ± 0.03	0.11 ± 0.02	0.13 ± 0.07	0.886^t^	0.991^M^
Zn (ppm)	1.74 ± 0.47	1.85 ± 0.57	0.503^M^	1.84 ± 0.25	2.00 ± 0.58	1.69 ± 0.54	1.71 ± 0.55	0.551^t^	0.851^M^

Plasma MT levels

Plasma MT-1A concentrations were determined according to a standard curve generated by the OD readings of the standards at 450 nm. The equation of the curve was found to be “y = 0.001 * x + 0.0304” with an R2 equal to 0.9831.

**Figure 4:**
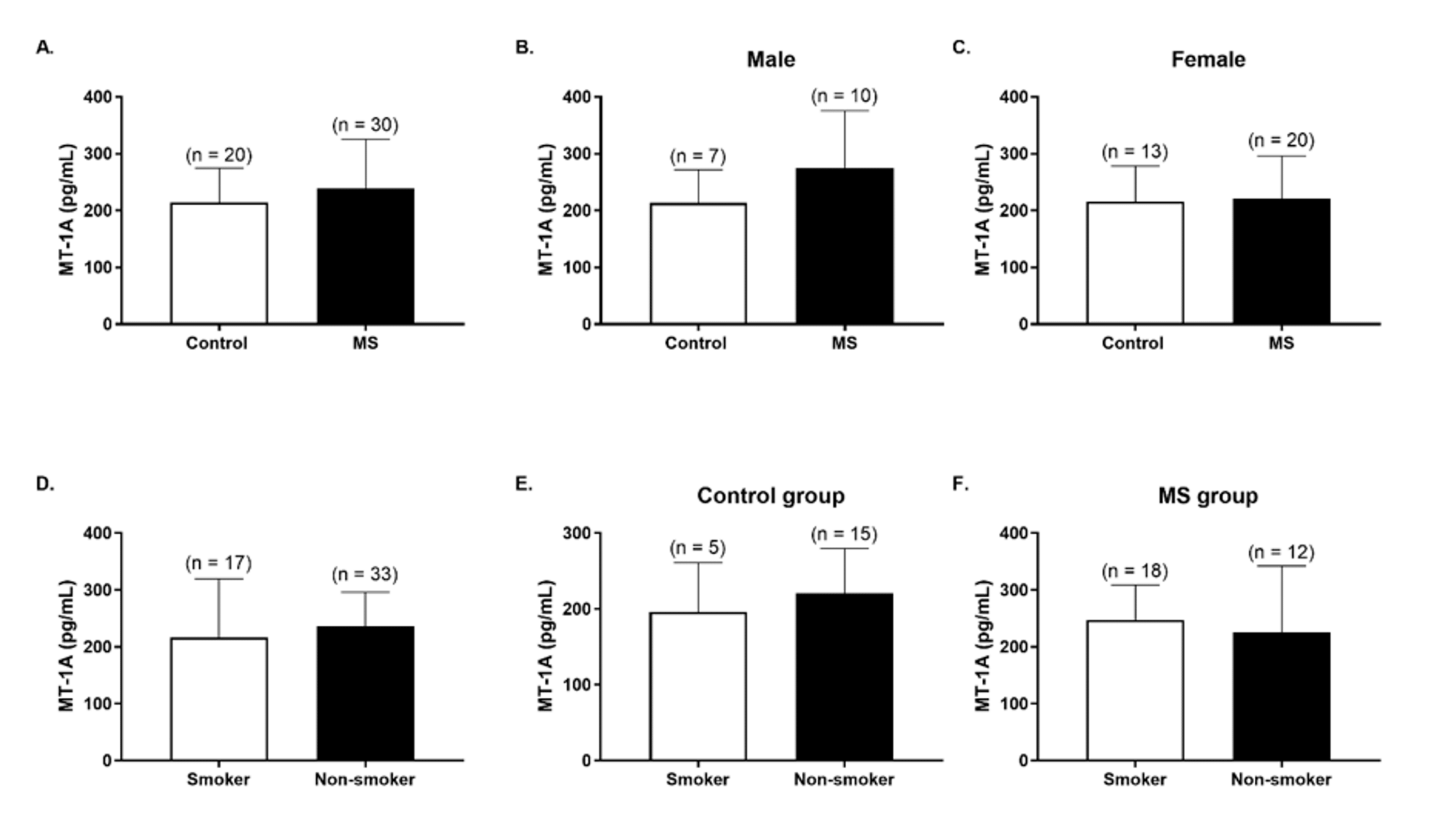
Plasma metallothionein-1a (MT-1A) concentrations in control and multiple sclerosis (MS) subjects (A), male subjects (B), and female subjects (C). Plasma MT-1A concentrations according to smoking status in the overall population (D), control group (E), and MS group (F) Data is presented as mean ± SD. Statistical analysis: unpaired Student’s t-test (p > 0.05 for all panels)

The mean MT-1A concentration was 214.50 ± 59.87 pg/mL in the control subjects and 238.60 ± 86.75 pg/mL in the MS patients (Figure 4A), with a slight difference between the groups (p > 0.05). Although not significant, slight differences in MT levels between the control and MS groups were found in both genders (in male subjects, control group = 213.50 ± 57.96 pg/mL vs. MS group = 274.80 ± 100.60 pg/mL; in female subjects, control group = 215.10 ± 63.21 pg/mL vs. MS group = 220.50 ± 75.23 pg/mL) (p > 0.05; Figures 4B, 4C).

In addition, differences in plasma MT-1A levels according to smoking status were not significant in the overall population, the control subjects, or the MS subjects (p > 0.05; Figures 4D-4F).

Plasma MDA levels

Plasma MDA concentrations were significantly higher in MS patients compared with control subjects (p < 0.0001; Table 3). Similarly, MDA concentrations were significantly higher both in male MS patients compared with male control subjects (p = 0.0024; Table 3), and in female MS patients compared with female control subjects (p = 0.0012; Table 3).

**Table 3:** Malondialdehyde (MDA) concentrations in the control subjects and multiple sclerosis (MS) group according to gender ^t^: Student’s t-test

	Control (mean ± SD)	MS (mean ± SD)	p-value	Male		Female		p-value	
				Control (mean ± SD)	MS (mean ± SD)	Control (mean ± SD)	MS (mean ± SD)	Male	Female
MDA concentrations	0.0180 ± 0.0039 µ mol/L	0.0258 ± 0.0063 µ mol/L	<0.0001^t^	0.0183 ± 0.0043	0.0265 ± 0.0047	0.0178 ± 0.0038	0.0255 ± 0.0070	0.0024^t^	0.0012^t^

Relationships between levels of heavy metal, essential metals, MT-1A, and MDA

Correlation analyses revealed no significant correlation between plasma MT-1A and blood As, Cd, or Pb levels, either in the overall population or in the control or MS patient groups (p > 0.05; Table 4).

**Table 4:** Correlation analyses between plasma metallothionein-1A (MT-1A) and blood heavy metal levels

MT-1A	Overall population		Control		MS	
	Pearson’s r	p-value	Pearson’s r	p-value	Pearson’s r	p-value
As	-0.03329	>0.05	0.2065	>0.05	-0.06753	>0.05
Cd	-0.07476	>0.05	-0.08646	>0.05	0.1501	>0.05
Pb	-0.02993	>0.05	0.05306	>0.05	-0.001891	>0.05

Plasma MDA concentrations in the overall population were found to show a significant moderate negative correlation with blood As levels (p = 0.0032; Table 5), while no significant correlations were observed either in the control subjects or in the MS patients (p > 0.05; Table 5). Moreover, plasma MDA concentrations in the overall population showed a significant weak-to-moderate negative correlation with blood Cd levels (p = 0.0095; Table 5), while no significant correlations were observed in the control and MS groups (p > 0.05; Table 5). Similarly, no significant correlations between blood Pb and plasma MDA levels were found in the cohort either as a whole or in the control and MS patient groups (p > 0.05; Table 5).

**Table 5:** Correlation analyses between plasma malondialdehyde (MDA) and blood heavy metal levels

MDA	Overall population		Control		MS	
	Pearson’s r	p-value	Pearson’s r	p-value	Pearson’s r	p-value
As	-0.4094	0.0032	-0.1968	>0.05	0.1953	>0.05
Cd	-0.3783	0.0095	-0.06983	>0.05	0.1772	>0.05
Pb	-0.2432	>0.05	-0.05647	>0.05	-0.1113	>0.05

No significant correlations between plasma MT-1A and blood As levels emerged either in the control group or in MS group subjects divided on the basis of smoking status (r = -0.7691; p > 0.05), while a weak negative but not a significant correlation between blood As and plasma MT levels in non-smoker subjects in the control group of patients (r = 0.33378; p > 0.05)

There was a weakly negative but not significant correlation between blood As and plasma MT levels in smoker subjects in MS patients (r = -0.2641; p > 0.05) and non-smoker MS patients, there was a very weak negative to no correlation, but not significant, between blood As and plasma MT levels (r = -0.05869; p > 0.05).

There was a strong, negative, and significant correlation between blood Cd and plasma MT levels in smoker subjects in the control group (r = -0.9157; p = 0.0290), while a moderate, positive, and significant correlation between blood Cd and plasma MT levels in non-smoker subjects in the control group (r = 0.6102; p = 0.0157). 

On the other hand, blood Cd and plasma MT levels in both smokers and non-smokers in the MS group were found in a very weak to positive to no correlation and these correlations were not significant (r = 0.1461 and r = 0.1586 for smokers and non-smokers subjects, respectively; p > 0.05).

There were no significant associations between blood Pb levels and plasma MT-1A levels according to smoking status in control subjects (r = -0.5543 and r = 0.08488 for smoker and non-smoker subjects; p > 0.05). However, while there were no significant associations between blood Pb and plasma MT-1A levels in smoker MS patients (r = 0.2612; p > 0.05), in non-smoker MS subjects, a moderately negative and statistically significant correlation was detected (r = -0.4701; p = 0.0490).

Finally, when evaluating correlations between essential metal levels and either MT-1A or MDA levels, no significant associations were found either in the control subjects or the MS group (p > 0.05). Moreover, no associations emerged between essential metal levels, MT-1A, and MDA levels when subjects were considered according to smoking status (p > 0.05).

Investigation of the relationship between plasma MT-1A and MDA levels revealed no associations whether considering subjects according to health status, gender, or smoking status (p > 0.05).

## Discussion

The exact etiology of MS is not completely understood, but both genetic and environmental factors contribute to its pathogenesis. Although heavy metals and MT have been suggested to play a role in MS pathogenesis, the studies are limited. In this study, we investigated the relationship between heavy metals (As, Cd, and Pb), and MT levels in MS patients.

Exposure to heavy metals, as well as organic solvents and chemicals, has been widely indicated to contribute to MS onset [34-36]. A 10-year-long epidemiological study conducted in Southwestern Taiwan revealed a relationship between elevated MS incidence and tap water use longer than 20 years that is related to As exposure [37]. A study conducted in Iran revealed three-fold higher serum As levels in RRMS patients compared to healthy controls. Moreover, oxidative stress and antioxidant activities were found to be elevated and decreased, respectively [38]. Moreover, blood As concentrations, as well as serum levels of S100B protein which is a biomarker for blood-brain barrier (BBB) dysfunction, were found to be elevated in RRMS patients [28]. In our study, RRMS patients had significantly lower As levels than the control patients which was independent of gender. As is known to accumulate not only in neurons but also in various organs including the heart, lung, liver, kidneys, and muscle, as well as in skin, nails, and hair [39]. Cardiovascular dysfunction [40] and respiratory dysfunction [41] are encountered in MS patients. Although these conditions in MS have been attributed to various mechanisms, it is not clearly known which is the major contributor. One of the possibilities is that cardiovascular diseases may be due to the As accumulation in the vascular system [42]. Similarly, As exposure has been suggested to cause impaired lung function [43,44]. It has been reported that As distributed throughout the body is promptly cleared from many tissues except for keratin-rich structures such as hair [45]. The blood half-life of arsenic is indicated to be shorter than six hours and in case the blood was not collected within two days for analyses, it may not be detectable [46]. The reason that we did not observe elevated levels of As in the blood of MS patients might be due to As accumulation in tissues.

Cd and Pb have also been suggested to be associated with MS. In our study, blood Cd and Pb levels were significantly lower in the MS patients than in the control subjects. Although these low Cd concentrations in MS patients were independent of gender, however, gender-based comparison of Pb levels did not show significant differences between the control and MS groups. Data on Pb, are controversial. One study, which also indicated elevated blood Cd levels, found no change in blood Pb levels [28]. Similarly, a study conducted in Iran revealed significantly higher serum Cd, but not Pb concentrations in MS patients compared with healthy control subjects [27]. Conversely, a study conducted in Taiwan revealed an association between soil Pb concentration and MS incidence (the authors did not investigate the relationship between blood Pb levels and MS incidence) [29]. A previous case-controlled study investigating blood Pb levels in MS patients showed that mean Pb did not differ significantly between control and MS groups [47]. Like As, both Pb and Cd [48,49] also accumulate in different tissues of the body. Most Cd is deposited in the kidneys (30%) and liver (30%), while the rest is distributed throughout the body, with a clearance half-life of 25 years [48]. The half-life of Cd in the blood has been estimated as 75 to 128 days, but this half-life primarily represents deposition in the tissues rather than clearance from the body [11]. Moreover, bone concentrations of Cd have been reported to increase in the human bone with age [50]. Pb, on the other hand, accumulates in different parts of the body including the brain and kidneys [49], although most accumulates in calcified tissues including bone and teeth [51,52]. In addition, the Pb half-life in blood and bone has been suggested to be 10-12 days and 10-30 years, respectively [51]. However, as with Cd, the half-life of Pb in the blood is an indication of the clearance of Pb from blood to bones [51]. Exposure to heavy metals, including Cd and Pb, occurs via different routes including habits, smoking, and occupation [53]. The lower blood Cd and Pb levels we observed in MS patients might be due to changes they have made in their lives, such as changing or quitting their jobs, altering their lifestyles, and quitting smoking. If this is the case, Cd and Pb might have already been cleared from the blood, while tissue Cd and Pb concentrations might still be high. As there are no imaging technologies able to detect tissue heavy metal content in a living person, without obtaining a biopsy sample from the tissue of interest, there is no precise way to demonstrate tissue concentrations of As, Cd, and Pb.

Gadolinium and gadolinium-based reagents have been used as imaging contrast agents in MS for some time [54-56]. Gadolinium has been reported to deposit in the MS brain early in the disease; however, its deposition was not found to be associated with disease severity [57]. Interference of gadolinium with most of the metals analyzed has been reported [58,59] and it has been suggested that blood samples for metal analyses should not be collected before 96 hours after gadolinium administration [46]. This interference might be another reason why we observed lower blood heavy metal levels in MS patients. In our study, all the MS patients were diagnosed with magnetic resonance imaging (MRI) scans and the timing of gadolinium administration for scanning purposes was not known at the time blood was collected.

Although single metals are thought to be toxic through specific pathways, the exact mechanisms of metal-induced toxicity are not fully understood. In addition to perturbations in metal metabolism, induced oxidative stress and neurodegeneration are also reported as a consequence of toxic metal exposure [60]. Cells in the central nervous system (CNS) have antioxidant mechanisms involving the glutathione system, superoxide dismutase, and catalase [61], but increased oxidant levels, as well as decreased antioxidant activity, lead to cell death [62]. Metal accumulation may induce reactive oxygen species (ROS) production and induce DNA damage, leading to apoptosis [63].

Humans are also exposed to redox-inert elements through the air, drinking water, and food. Even at low concentrations, these elements exert toxicity through various mechanisms, for example by depleting glutathione or binding to proteins [64], and they have no biological benefits. Lead, which is a redox-inert element, is widely used in industrial applications thanks to its physicochemical properties [65]. However, it can persist in the environment for long periods and is toxic to both animals and humans. Lead damages cells by deactivating certain enzymes and antioxidant sulfhydryl pools, disrupting the absorption of important trace minerals and thus promoting oxidative stress [66]. Pb toxicity, which is mainly diagnosed by increased blood levels, causes various diseases including neurological disorders. Lead exposure has been widely linked with amyotrophic lateral sclerosis (ALS) and ALS-like conditions [67,68], and a possible link with poor cognitive performances has also been reported in clinical studies [69,70]. However, the relationship between blood Pb levels and Alzheimer’s disease (AD) remains controversial. Giacoppo et al. found a positive correlation between AD and increased blood Pb levels [71], whereas other studies did not find any significant increase in Pb concentrations [72-76].

MTs are induced by exposure to heavy metals and play a role in protecting the organism against heavy metal-induced toxicity [77]. In rats, it has been suggested that plasma MT levels can be a supportive biomarker of Cd toxicity [78]. MTs have been shown to be protective against As [79]. Moreover, the binding of Pb to MTs has been reported in various studies [80]. Roles and expression patterns of MTs have been studied in various neurodegenerative diseases [81]. However, data on MT levels and their roles in MS are limited and controversial. In our population, both overall and divided by gender, no significant differences in plasma MT levels were found between control subjects and MS patients. In EAE mouse models, MT-1 and MT-2 levels, but not MT-3 levels, were found to be significantly elevated in the CNS [25]. In addition, MT-1 and MT-2 deficiency led to altered inflammatory response, oxidative stress, and apoptosis, resulting in higher neuronal damage in EAE mice [82]. In a human study in which MT-1 and MT-2 protein expression was investigated in the brains of three deceased MS patients and two controls, both active and inactive lesions revealed elevated immunoreactivity of MT-1 and MT-2 in all MS cellular infiltrates with a similar expression pattern to those observed in the EAE model [82]. However, another study that investigated the MT-1 and MT-2 levels in the brain and blood samples from EAE mice during the progression of the clinical phenotype failed to show any significant alteration [83]. On the other hand, MT-2, but not MT-1, has been shown to alleviate the neuroinflammation and clinical course in EAE mice [84].

The relation of essential metals in MS is not fully clarified. There is conflicting information on the relation between Fe, Cu, and Zn in MS. In different studies, Fe levels were significantly lower in MS patients [85-87]. A systematic review and a meta-analysis concluded lower Fe levels in MS patients [88]. In our study, Fe levels were significantly lower in MS patients.

Data on the relationship between Cu levels and MS patients are various. In a study, they found significantly lower levels of Cu in MS patients [85], while in another study Cu levels were significantly higher in MS patients [86]. In a meta-analysis, Cu was not significantly associated with MS [88]. Our results showed Cu was significantly lower in MS patients.

Zn levels in different studies vary. In a study, Zn levels were not significant but lower [85], while in another study Zn levels were higher but not significant in MS patients [87]. In another study, Zn levels were significantly lower in MS patients [86]. A meta-analysis reported lower Zn levels in MS patients [88]. We found significantly lower Zn levels in MS patients in our study.

In our study, As, Cd, and Pb levels were found to be significantly lower in the MS patients than in the control subjects; MT levels, on the other hand, were higher in the MS patients, this difference was not statistically significant and possibly due to the limited number of subjects included in our study. It is not very easy to explain why the heavy metal levels in MS patients are lower than in control subjects. From this point of view, these questions can be asked. Are heavy metal kinetics in MS patients different from normal subjects? Are the body distribution of heavy metals in MS patients different from normal subjects? New thinking is necessary to understand this situation.

Limitations of the study

Our study was conducted in only one center and a limited number of patients. More studies with a larger cohort in a randomized, multi-centered, double-blind design will be more helpful in further understanding the relationship between MS and heavy metals.

## Conclusions

In this study, which was conducted in a small cohort living in an industrial region in Kocaeli, heavy metals were not found to be related to MS. However, our results suggest that MT levels play an important role in MS, in line with what is found in other neurological disorders. Randomized, double-blinded, multi-centered clinical studies in larger cohorts are needed to further understand the relationship between heavy metals and MT levels in MS patients. Moreover, studies designed to investigate the role of heavy metal levels should be planned in such a way that blood samples are collected before the first MRI before diagnosis. Thus, all interventions, such as lifestyle changes, that will interfere with further analyses may be excluded. After the diagnosis of MS by MRI scans, patients diagnosed with MS and those not diagnosed with MS may be allocated as patient and control groups, respectively.
